# Temporal–spatial variability of grazing behaviors of yaks and the drivers of their intake on the eastern Qinghai-Tibetan Plateau

**DOI:** 10.3389/fvets.2024.1393136

**Published:** 2024-06-11

**Authors:** Xiaoqian Yang, Umar Daraz, Jianguo Ma, Xingxin Lu, Qingshan Feng, Huaide Zhu, Xiao-Bo Wang

**Affiliations:** State Key Laboratory of Herbage Improvement and Grassland Agro-Ecosystems, College of Pastoral Agriculture Science and Technology, Center for Grassland Microbiome, Lanzhou University, Lanzhou, China

**Keywords:** grazing behaviors, intake, yak, Qinghai-Tibet Plateau, grassland

## Abstract

**Introduction:**

Grassland-livestock balance is an important principle of sustainable development of grassland livestock production and grassland ecosystem health. Grassland degradation becomes more serious at global scales and especially at the area that is sensitive to climate change and human activities. Decreases in pasture biomass and shifts in plant community composition in degraded grasslands can largely affect grazing behaviors of livestock. Up to date, however, it is unclear that whether livestock behaviors change across spatial and temporal scales and what key factors are to shape observed behavioral patterns of livestock.

**Methods:**

Here, yak behaviors including grazing, rumination and walking on the eastern Qinghai-Tibetan Plateau (QTP) were monitored by a continuous visual observation, to investigate temporal and spatial variations of grazing behavior of yaks (Bos grunniens); based on the data from public database in the past 18 years, a meta-analysis was conducted to examine the main factors that affect grazing behaviors and intake of yaks.

**Results:**

We showed that grazing behaviors of yaks differed significantly within hours, among hours of each day and among days as well as across different observation sites. Intake rate of yaks was higher in the morning than in the afternoon, but walking speed showed an inverse trend compared with intake rate. Resting, altitude, the mean annual precipitation (MAP), the mean annual temperature (MAT), forage ash, yak age and season were the main predictors for yak intake, and forage and yak individual characteristics had direct effects on grazing behaviors and intake of yaks.

**Discussion:**

The findings confirm that grazing behaviors of yaks can vary even at small temporal scales and regional scales, which is closely related to the shift in forage quality and biomass caused by environmental changes. The study suggests that multiple factors can be responsible for the variation in livestock behaviors and shifts in behavioral patterns may consequently lead to positive or negative feedback to grassland ecosystems through plant-animal interactions.

## 1 Introduction

Livestock are key components in a natural grassland ecosystem and play essential roles in regulating grassland ecosystem health and services. Traditionally, since the land use in the grassland is associated closely with animal husbandry, many grasslands worldwide have heavily relied on grazing for hundreds of years for the purpose of satisfying increasing demands for products (1, 2). However, negative effects of anthropogenic activities on rangeland ecosystems are being intensified due to grazing-induced shifts in ecosystem structure, functioning and stability (3, 4). Particularly, at local and regional scales, changes in plant community composition such as decreased numbers of palatable forage in degraded grasslands, have affected heavily on the behavior of livestock, especially for large herbivores (5). Livestock behaviors can also produce an impact on above-belowground ecological processes such as plant succession and nutrient cycling via grazing, treading and excreta return (4, 6). Understanding the changes of livestock behaviors can provide important parameters for modeling livestock intake and improve predictions of grassland ecosystem health, and consequently achieving sustainable management of livestock grazing in grassland ecosystems (6, 7).

Grazing, ruminating, and walking are the three primary activities carried out by livestock. Generally, free-ranging livestock spend much energy on grazing and walking. It is reported that ruminants spend 90–95% of their daily time grazing, ruminating, and resting in the pasture-based system (8). This may result in a significant increase in the amount of energy that is consumed (6, 9). The higher consumption of energy associated with physical activity may raise animals’ maintenance energy requirements and reduce the energy availability for growth and development (10). The daily intake capability of forage is thus dependent greatly on the amount of time spent grazing and the rate of forage consumption throughout that period. Daily consumption of forage is proportional to the number of bites per unit time (bite rate) and the mass of forage consumed per bite (bite mass) (11). Rumination is the behavior utilized by ruminants after grazing, which is crucial for feed breakdown because it raises the specific gravity of forages, shreds plant tissue coverings, and provides more of the forage surface area to the rumen microbiota. Some studies have shown that grazing and ruminating behaviors of ruminants are cyclical (12) and can change based on the forage quality and types, environmental conditions, individual characteristics of livestock, and different grazing intensities (13–16).

Livestock behaviors in natural grassland vary temporally and spatially, depending strongly on resource availability and changing environments that they live (17, 18). Due to the energy and metabolic demands, vegetable dynamics is mainly responsible for livestock behaviors. Plant composition and distribution have been well demonstrated to vary largely over space and time (5, 19). One of the important consequences of such variations is to lead to temporal and spatial variations in livestock behaviors. For example, ruminants’ grazing behavior often changes with a shift in herbage biomass and pasture nutritional quality (20). In a ranch with abundant vegetation, ruminants generally gather around the areas that have good quality forage (21, 22). In a ranch with spatially homogenous resources, the herbage resources are often utilized through selective grazing by ruminants to meet their nutrient demand and energy supply so that plant community shows a mosaic pattern (23). As a result, the factors that influence plant growth and physiological activities, including climate, altitude and soil conditions etc. can affect directly and indirectly the grazing livestock behaviors. Soil spatial heterogeneity strongly influences the growth and physiology of individual plants (24, 25), the dynamics of plant populations (26) and interspecific interactions (25), and plant community composition (27). Alternatively, temporal and spatial variations in climatic conditions, such as the inter- and intra-annual variability of precipitation and temperature have also led to significant shifts in the plant community characteristics (28, 29). However, up to date, our understanding about how livestock behaviors change across spatial and temporal scales and what factors are main drivers that shape these behavioral patterns of livestock remains very limited.

The Qinghai-Tibetan Plateau (QTP) is the largest grassland ecosystem in Eurasia, where the yak is the most important livestock grazing on the highlands (30). It is estimated that there are over 13.3 million domesticated yaks (*Bos grunniens*) that freely graze in this area (31, 32), ranging from the extensive grassland of the QTP to regions surrounding the Himalayan Mountains. Throughout the year, yaks are grazed on natural pastures of traditional ranches without the need for supplementary feeding (33). Therefore, the yak can adapt well to variations of plant community composition, biomass, and abiotic environments induced by seasons via an adjustment of their own grazing behaviors (34, 35). However, only a few studies have examined the behavioral patterns of grazing yaks of the QTP (36, 37). A recent study on the QTP has even shown that yak behaviors can vary throughout a day (38), but more field-based survey is needed to understand whether the yak behavior can vary at temporal and spatial scales and what factors are crucial to affect yak behaviors.

In this study, we hypothesized that (i) yak behavior would vary at both short temporal scales (hour, day and week) and at ranch scales with similar grazing intensity; and (ii) grazing behaviors of yak would be also strongly influenced by multiple factors including climate, season, altitude and forage characteristics, which would consequently affect intake of yak on the QTP. To test these hypotheses, first, three main yak behaviors including grazing, rumination and walking were continually monitored for 1 week at Maqu Research Station on the QTP; secondly, we selected four ranches nearby Maqu with similar grazing intensity to investigate spatial variations in grazing behavior; thirdly, we conducted a meta-analysis for yak behaviors based on a search of papers that were achieved in public database over the past 18 years, to examine the main factors that affect yak behaviors and intake. We aim to reveal the temporal and spatial variations of yak behaviors and figure out what factors are main drivers that shape observed behavior patterns of yak on the QTP.

## 2 Materials and methods

### 2.1 Study areas and animals

All trial procedures strictly followed rules and regulations of the Experimental Field Management protocols (files 2010–1 and 2010–2) of Lanzhou University and were approved by the Animal Ethics Committee of Lanzhou University.

**Figure 1:**
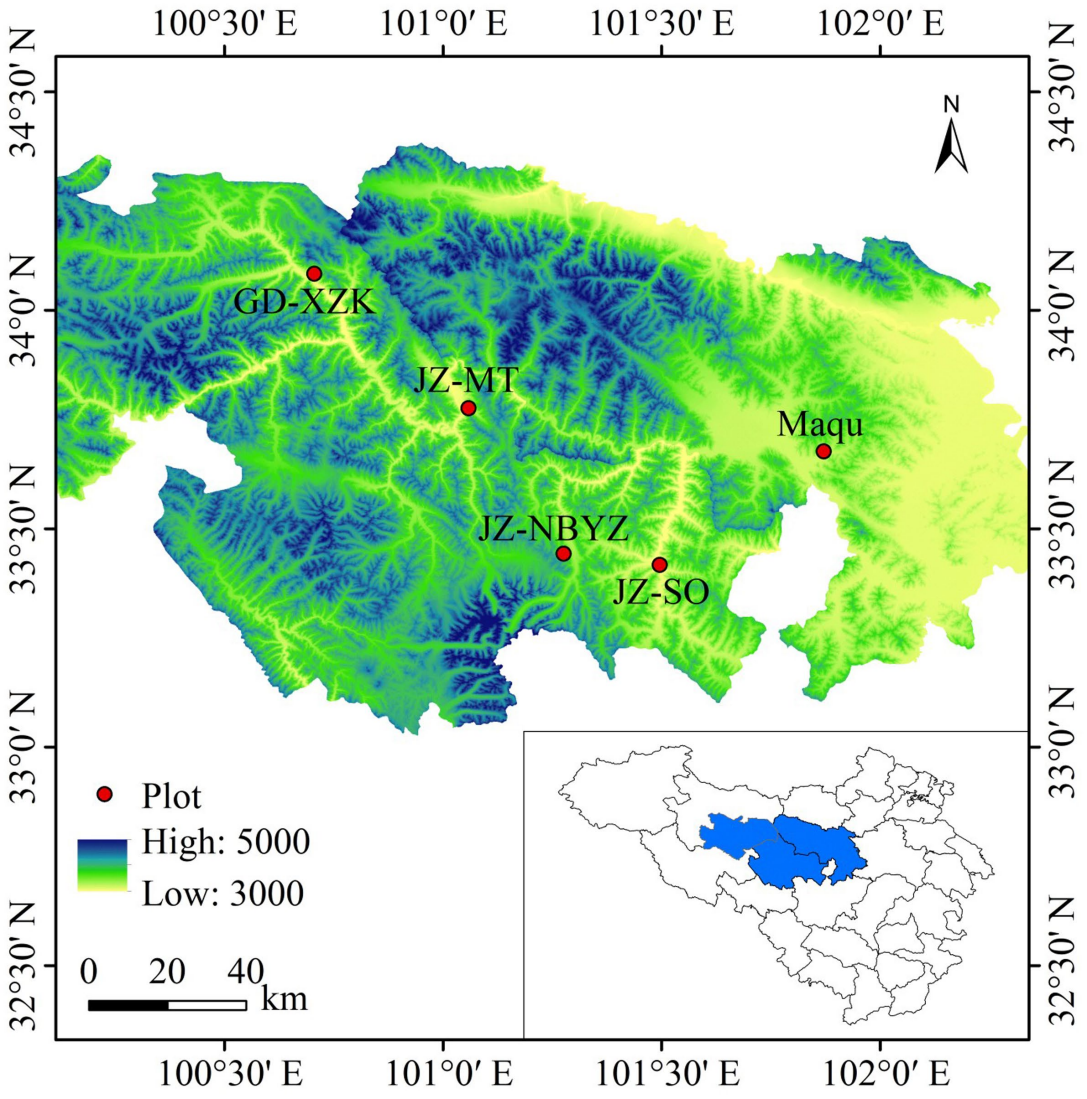
The location of five survey sites with showing geographical coordinates and altitude on the eastern Qinghai-Tibetan Plateau, China. The sites of Maqu and JZ_MT are located in Maqu and GD_XZK Country, respectively. The sites of JZ_SO, JZ_NBYZ are located in Jiuzhi County. The graph was generated using ArcGIS (version 8.0).

Yak behaviors were monitored by visual observation during 12 July to 20 July near the peak of the plant growth at Maqu, Jiuzhi and Gande County on the eastern QTP of China (Figure 1). The three locations are typical areas where yaks are raised on the QTP, where the mean annual temperature (MAT) is 1.2°C and mean annual precipitation (MAP) is 620 mm. A total of five sites with yak grazing in the three locations were selected; one site (Maqu) was located in Maqu Country (101°52′E, 33°40′N) with an altitude of 3,536 m a.s.l., three sites (JZ_SO, JZ_NBYZ and JZ_MT) were located in Jiuzhi Country (101°29′E, 33°25′N; 101°16′E, 33°26′N; 101°3′E, 33°46′N) with an altitude of 3,650 m, 3,888 m and 3,782 m a.s.l., and one site (GD_XZK) was located in Gande Country (100°42′E, 34°5′N) with an altitude of 3,853 m a.s.l. The main grassland type in these regions is typical alpine meadow, with *Kobresia capillifolia*, *Carex thibetica*, *Elymus nutans*, *Poa pratensis*, *Stipa aliena*, *Anemone rivularis* var. *flore-minore*, *Halenia corniculata*, and *Ligularia virgaurea* being dominant plant species. In each site, yaks were free to be grazed with a grazing intensity of 1.5–2 head of yak/ha from 9:00–17:00 every day, housed in shelters overnight and did not receive any supplementary feed during the experimental period.

### 2.2 Grazing behaviors

We carried out a continuous 6-day monitor of yak behaviors at Maqu. To minimum the effect of differences in individual characteristics on the grazing behaviors, yaks were selected based on their body weight (BW) and age across all survey sites in this study. Thus, a total of 18 yaks with BW of around 200 kg and an age of 3 years were selected as survey objects. Yak behaviors including the intake rate (bites per min), walking speed (steps per min) and rumination frequency per min were visually recorded by independent observers who were divided into two groups (three independent observers within each group). All participants have rich experiences in monitoring yak behaviors and were trained well before the data were collected. Yak behaviors including intake rate (bites per min), walking speed (steps per min), rumination frequency (per min) were recorded every 10 min within each hour between 9:00 and 17:00 (9:00–10:00, 10:00–11:00, 11:00–12:00, 14:00–15:00, 15:00–16:00, and 16:00–17:00). Each behavior was calculated based on the average of obtained data from all observations of two groups. Bite rate was determined as the time that animals spent taking 60 bites. If the time between bites was longer than 15 s the measurement was canceled and started over (39). To examine ranch-scale variations in grazing behavior, we also carried out a similar observation of yak behaviors for the other four sites from 18 July to 20 July.

### 2.3 Meta analysis

A meta-analysis was conducted to assess the main factors that affect grazing behaviors and intake of yaks on the QTP. The related references published on the QTP were identified based on a search of keywords including “grazing behavior” or “behavior” or “feed intake” or “intake” and “yak” during the past 18 years (2004–2021), which was recorded in the online database of WoS (Web of Science, http://www.webofknowledge.com/) and CNKI (China National Knowledge Infrastructure, https://www.cnki.net/). The geographic coordinates in each reference were uniformly converted by the online software.^1^ The data sets of each graph in the reference were extracted by online software Web Plot Digitizer.^2^ Ultimately, a total of 15 articles were collected in which 43 observations were used for subsequent analysis (40) (Supplementary Figure S3 and Supplementary Table S1), including altitude, climate (MAP, MAT), grazing behavior [intake, walking, resting and rumination (Rum)], season (cold and warm), forage characteristics [ash (ASH), crude protein (CP), neutral detergent fiber (NDF) and acid detergent fiber (ADF)], individual characteristics of livestock (age and weight) and grazing intensity (GI).

### 2.4 Statistical analysis

All statistical analysis and figure generation were performed in R (v.4.0.3). The bar charts and box plots were generated using the geom_boxplot, geom_bar, and geom_smooth functions of the ggplot2 package. One-way analysis of variance (ANOVA) with *post hoc* tests were used to test the significant differences in the intake rate, rumination and walking among different times and survey sites. A quantile-quantile plot (Q–Q plot) was carried out to assess whether the residuals of each variable in the collected data sets are normal distributed by qqPlot function. We used Random Forest model to determine which variables in the data sets are the main predictors of the intake rate. Since the randomForest package of R statistical software does not provide significance of the prediction variable, the significance of each prediction variable to the response variable was assessed by using the “rfPermute” package. Structural Equation Modelling (SEM) was performed using IBM SPSS Amos 24.0 software to further evaluate the direct and indirect relationships between prediction variables selected by Fandom Forest model and the intake rate. Before doing SEM, all the data were standardized and carried out principal component analysis (PCA) for each module including climate, forage characteristics, grazing season, grazing behavior and individual characteristics of yak with vegan package. We only chose the first principal component (PC1) of each module as the variables in the SEM. The best fit of SEM was assessed by the chi-square test (*p* < 0.001) and RMSEA.

## 3 Results

### 3.1 Temporal and spatial variations of grazing behavior

**Figure 2:**
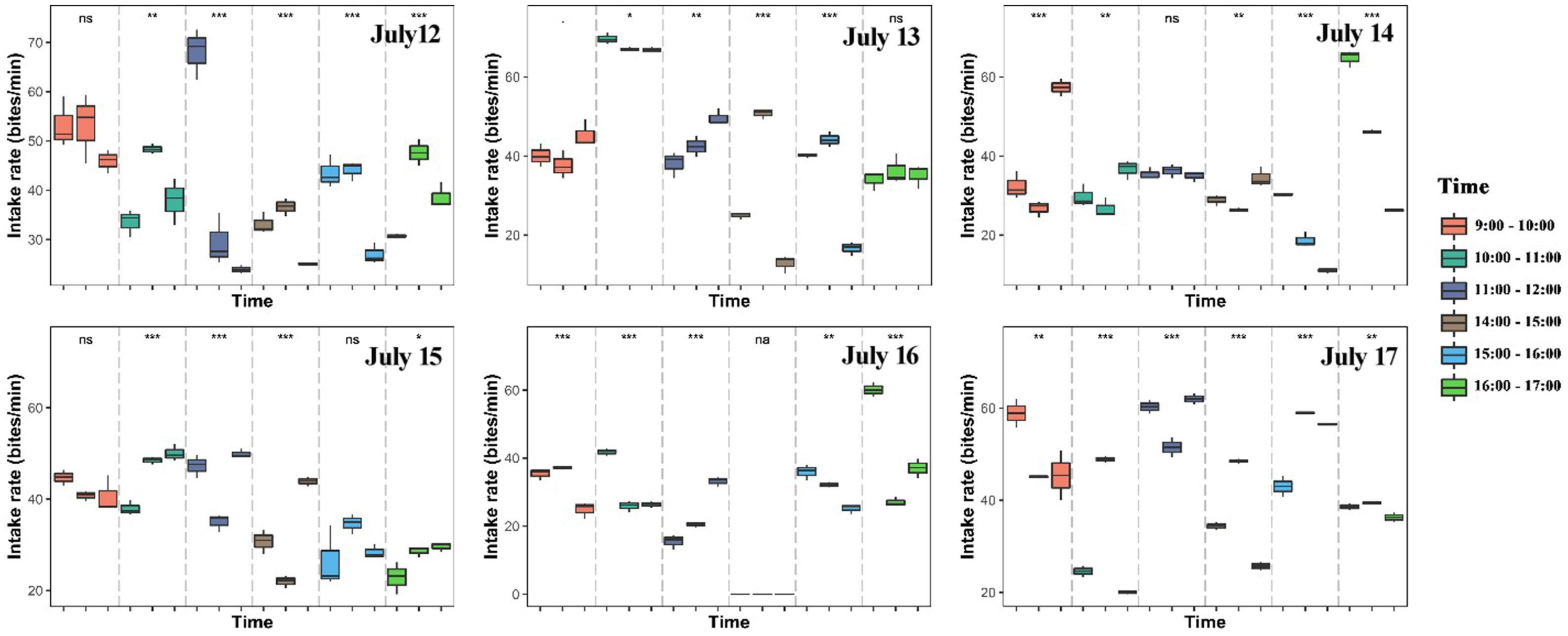
Differences in intake rate (bites/min) of yaks within hours (9:00–10:00, 10:00–11:00, 11:00–12:00, 14:00–15:00, 15:00–16:00, and 16:00–17:00) of each day. Asterisk indicates significant difference at ^*^p < 0.05, ^**^p < 0.01, ^***^p < 0.001.

**Figure 3:**
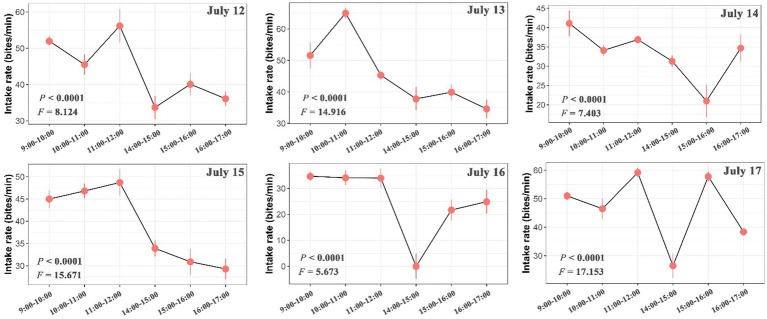
Differences in intake rate (bites/min) of yaks among hours of each day. The bars represent the standard errors. The statistically significance was tested by ANOVA at *p* < 0.05.

The number of bites was observed and counted three times in total within each hour (10 min per time in all animals). We found large variations in the intake rate of yaks within hours, among hours of each day and among days (Figures 2, 3; Supplementary Figure S1). The intake rate differed significantly within each hour of each day during 12 July to 17 July (*p* < 0.05) (Figure 2). Likewise, intake rate also differed significantly among hours of each day (*p* < 0.001) (Figure 3) and among days (*p* < 0.001) (Supplementary Figure S1). The intake rate generally reached maximum at 11:00–12:00 and decreased to minimum at 14:00–15:00 in each day (Figure 3).

**Figure 4:**
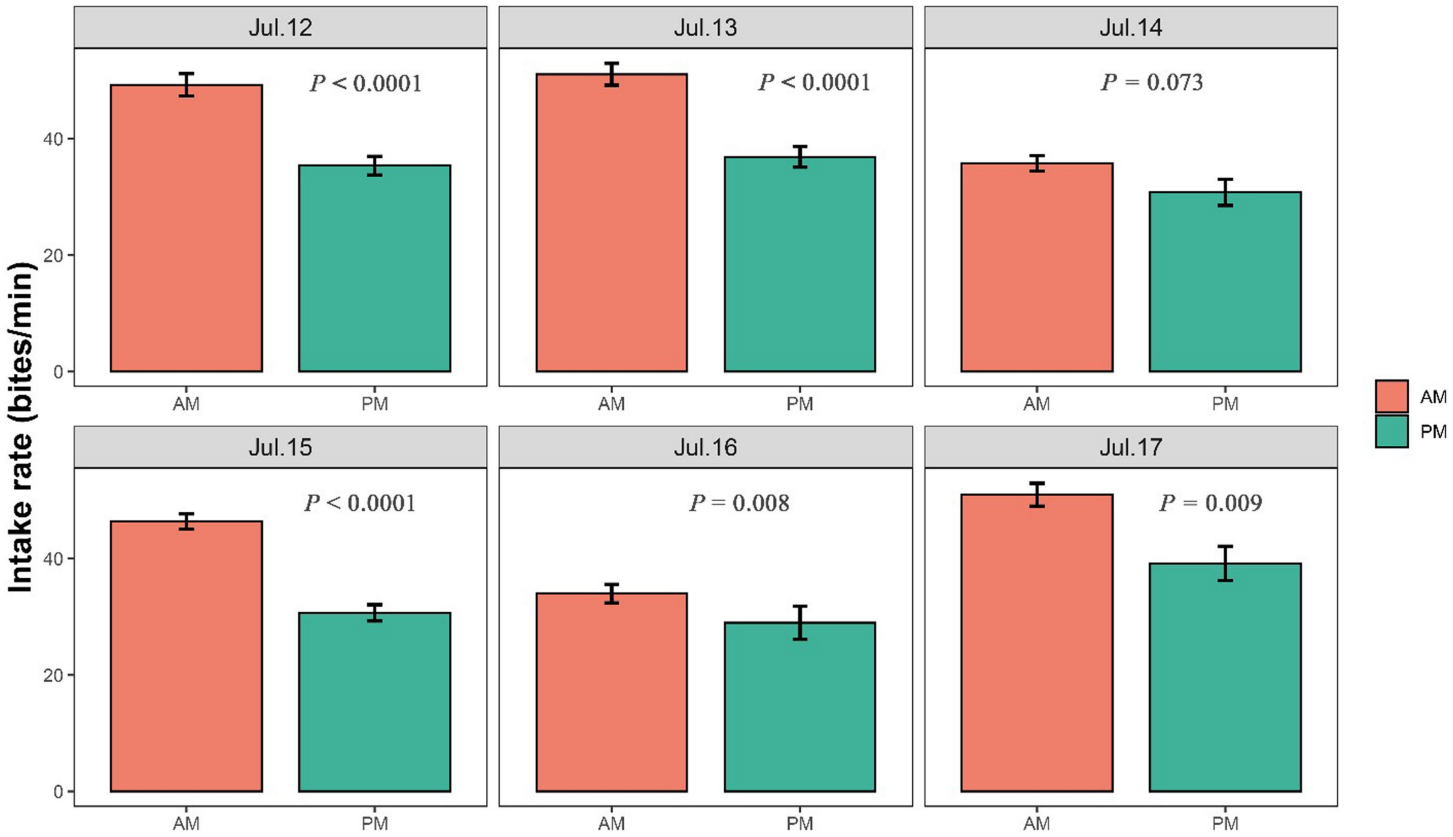
Differences in intake rate (bites/min) of yaks between in the morning and in the afternoon. The bar charts display standard errors. The statistically significance was tested by ANOVA at *p* < 0.05.

**Figure 5:**
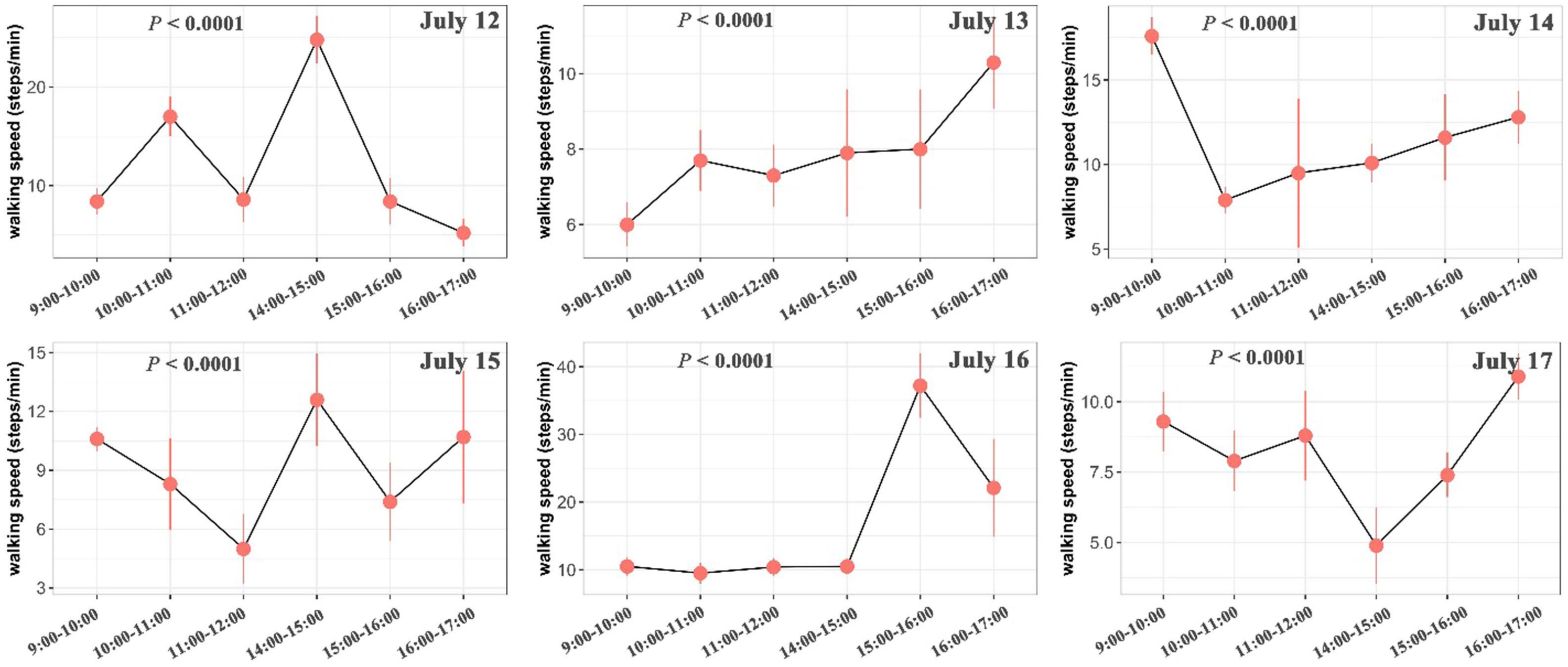
Differences in walking speed (step/min) of yaks among hours of each day. The bars represent the standard errors. The statistically significance was tested by ANOVA at *p* < 0.05.

The intake rate was significantly higher in the morning than in the afternoon in July 12 (*p* < 0.001), July 13 (*p* < 0.001), July 15 (*p* < 0.001), July 16 (*p* = 0.008) and July 17 (*p* = 0.009), but was marginally significantly higher in July 14 (*p* = 0.073) (Figure 4). Walking speed of yaks showed an inverse trend compared with the intake rate, with showing higher speed in the afternoon than in the morning (Figure 5; Supplementary Figure S2). Walking speed of yaks also differed significantly among hours of each day (*p* < 0.001) (Figure 5) and among days (*p* < 0.001) (Supplementary Figure S2).

**Figure 6:**
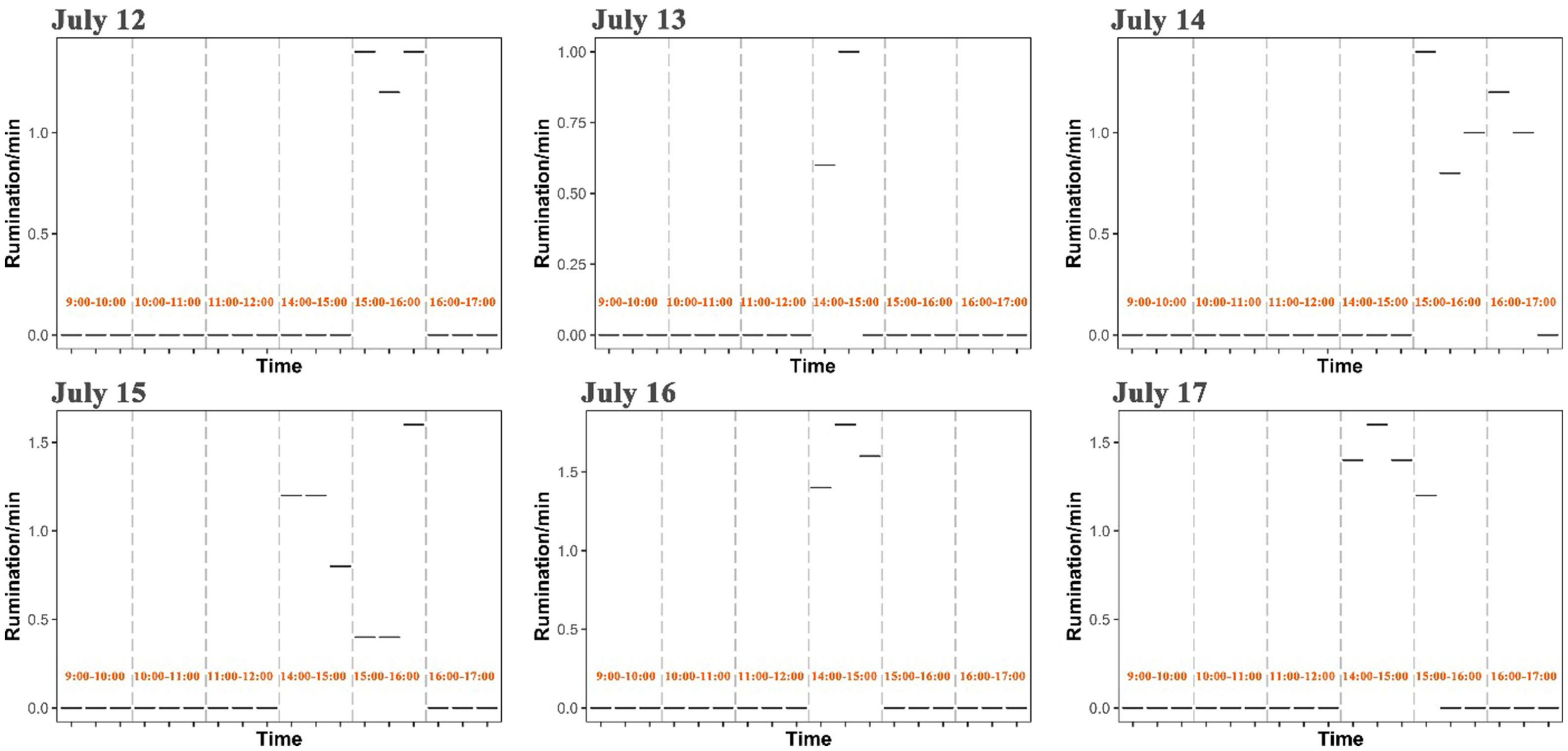
Distribution of rumination time of yaks across each day.

**Figure 7:**
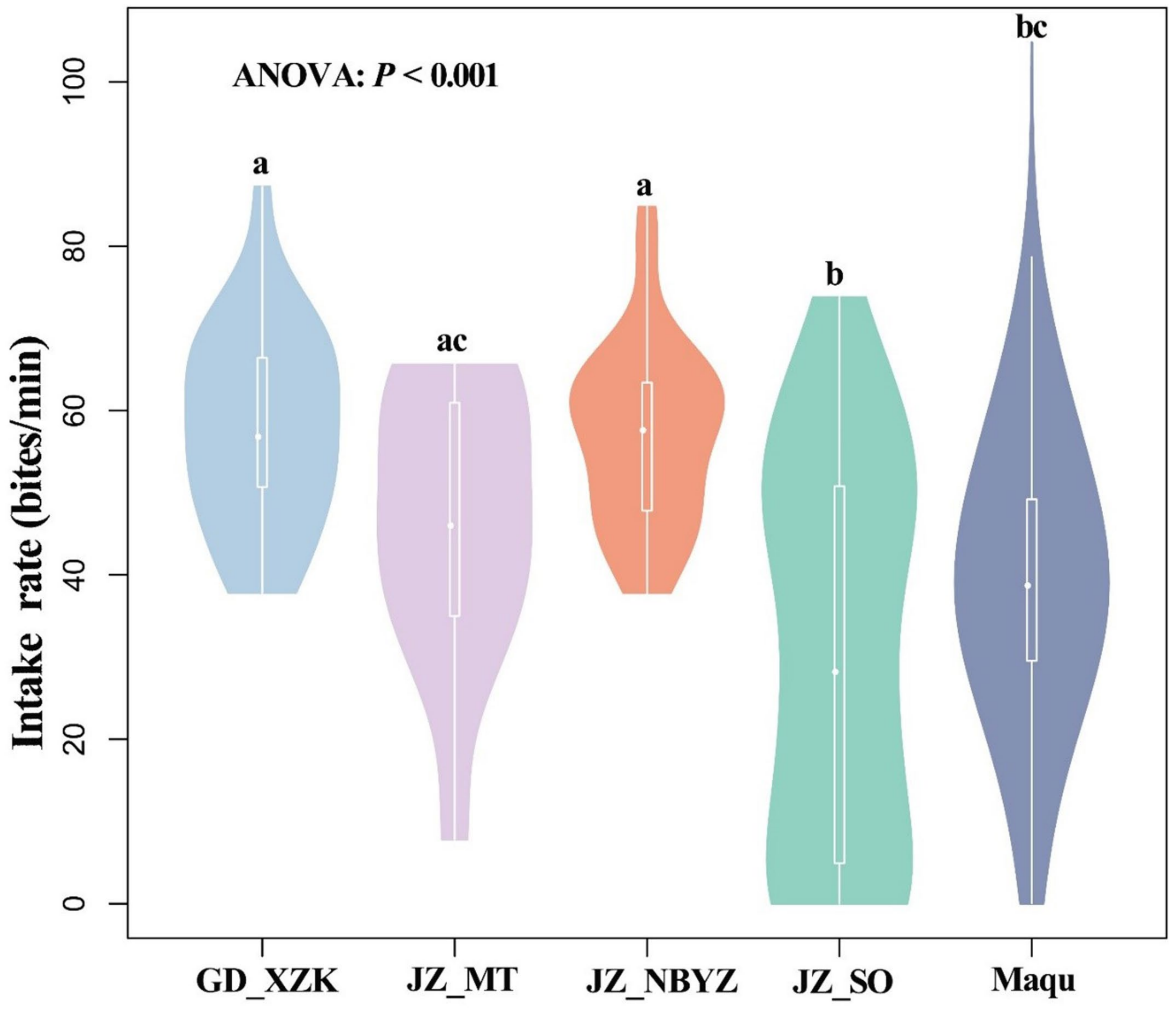
Violin diagram of the differences in feed intake (bite/min) of yaks across different observation sites of Qinghai-Tibetan Plateau. Lowercase indicates statistically significant differences at *p* < 0.05 by ANOVA.

Rumination of yaks generally occurred at 14:00–16:00 in the afternoon with a frequency of 0.4–1.8 per minute, but rumination time was observed to vary in each day from July 12 to July 17 (Figure 6). Based on an observation across five survey sites, the intake rate, walking speed and rumination of yaks showed spatial variations and differed significantly among sites (*p* < 0.001) (Figure 7 and Supplementary Figure S4). The intake rate of yaks in GD_XZK and JZ_NBYZ was significantly higher than in JZ_SO and Maqu (*p* < 0.05) (Figure 7). The intake rate of yaks in JZ_MT was only significantly higher than in JZ_SO (*p* < 0.05) (Figure 7).

### 3.2 Meta-analysis for grazing yaks on the QTP

**Figure 8:**
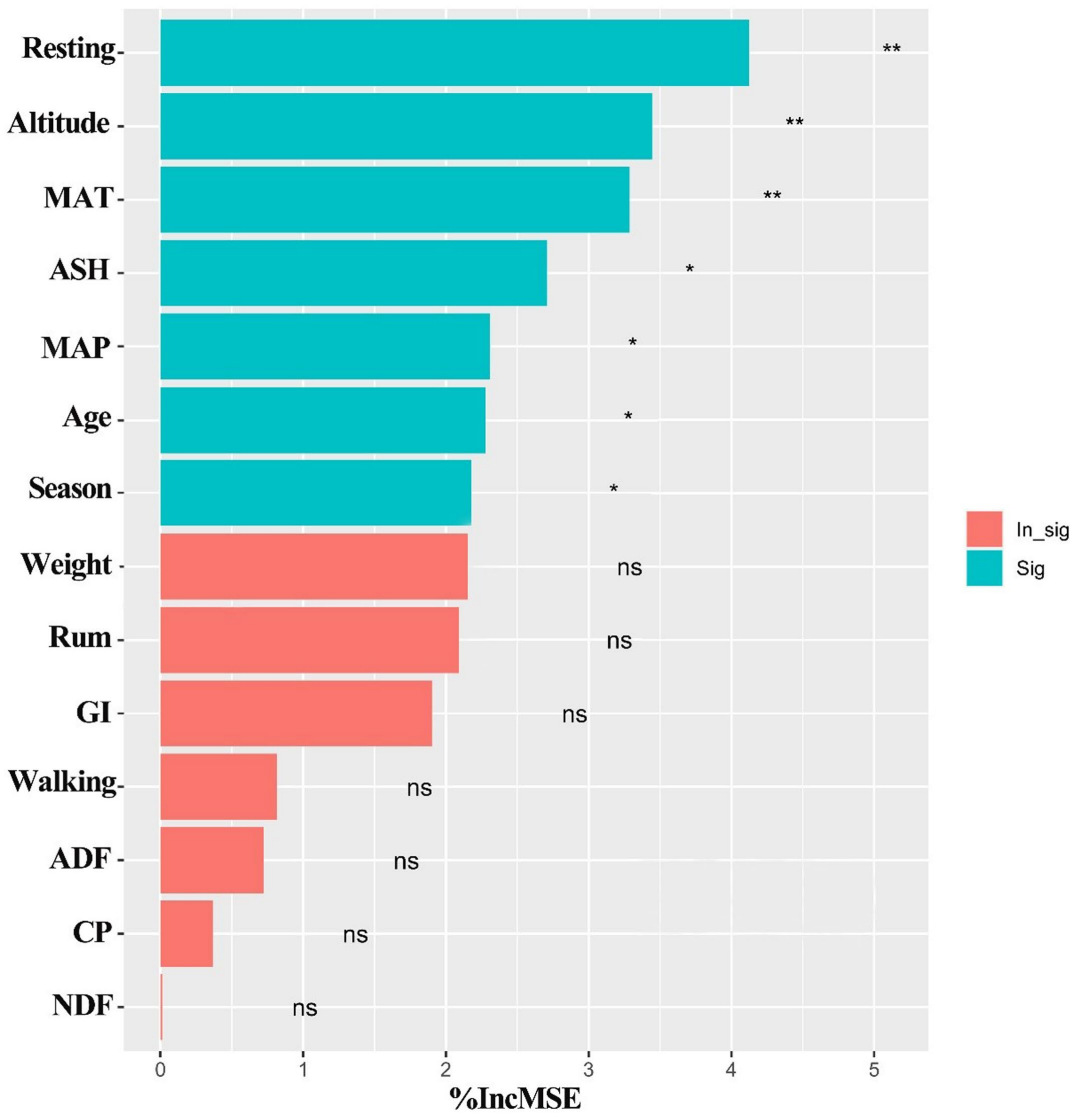
Random Forest model for evaluating significant factors affecting yak intake on the Qinghai-Tibetan Plateau, based on the data from public database during the past 18 years. Significance levels of each predictor are ^*^p < 0.05 and ^**^p < 0.01. ASH, Ash; MAT, mean annual temperature; GI, grazing intensity; CP, crude protein; ADF, Acid detergent fiber; Rum, rumination; NDF, neutral detergent fiber; MAP, mean annual precipitation; Age, yak age; Season, livestock season; Weight, yak weight.

**Figure 9:**
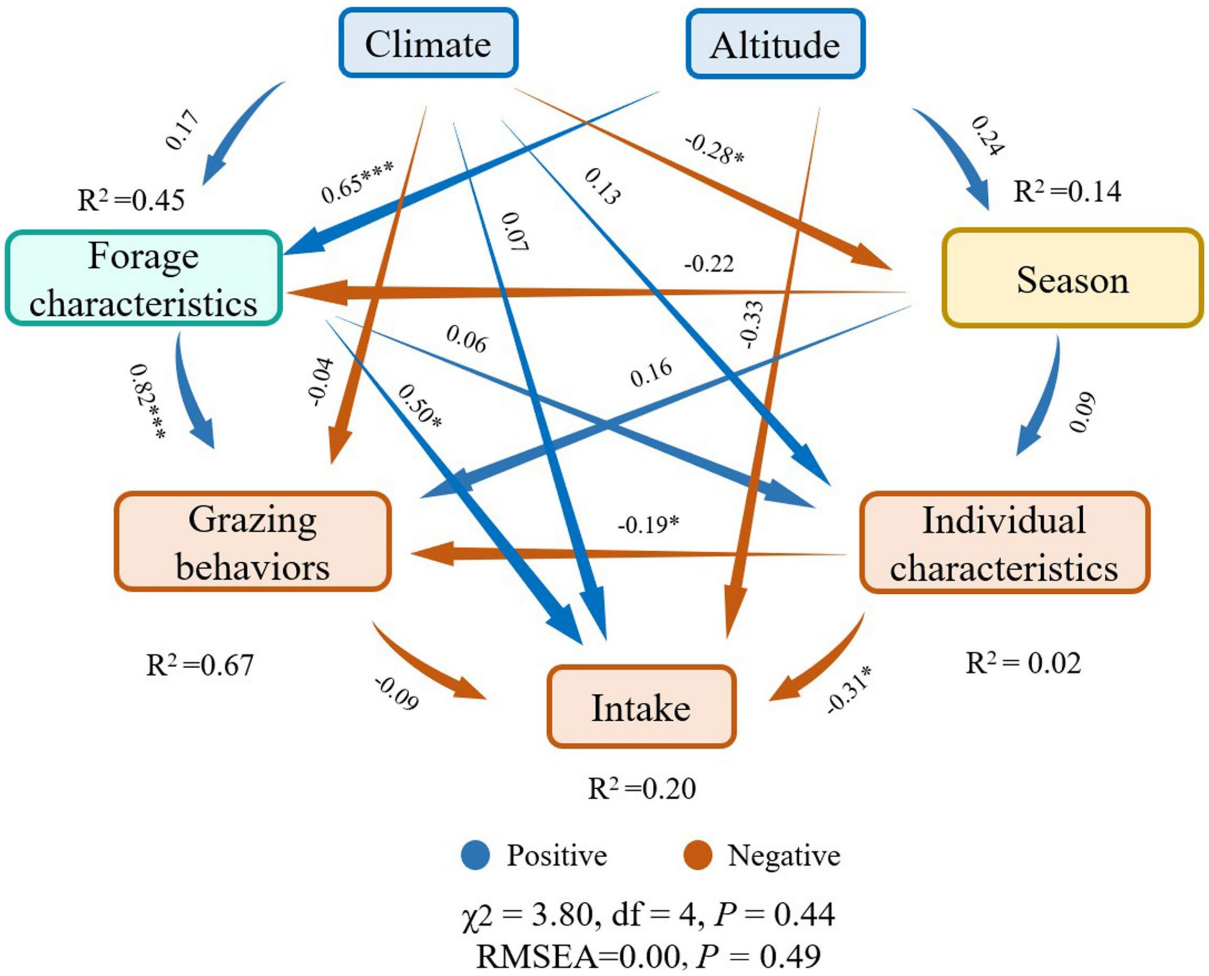
Structural equation model describing the effects of multiple factors including climate, altitude, season, forage characteristics, grazing behaviors and individual characteristics on yak intake. Numbers adjacent to arrows are indicative of the effect size of the relationship. Arrows reflect causality with blue (positive) and red (negative). *R*^2^ denotes the proportion of variance explained. Significance levels of each predictor are *p* < 0.10, ^*^p < 0.05, and ^**^p < 0.01. In all cases, there was a non-significant deviation of the data from the model (χ^2^ = 3.80, df = 4; *p* = 0.44; RMSEA = 0.00; *p* = 0.49). ***p < 0.001.

Among the variables we obtained from online database, resting, altitude, MAT, ASH, MAP, livestock age and season were found to be the most important predictors for the intake of yaks (*p* < 0.05) (Figure 8), while other variables including weight, grazing intensity (GI), walking, ADF, CP, rumination (Rum) and NDF were not significant predictors for the intake rate of yaks (*p* > 0.05) (Figure 8), based on the analysis of Random Forest model. Structural equation model (SEM) showed that climate, altitude, forage characteristics, season, grazing behaviors, and livestock individual characteristics had direct or indirect effects on the intake (Figure 9). Altitude did not significantly affect the intake directly (*λ* = −0.33; *p* > 0.05), but significantly positively affected forage characteristics (*λ* = 0.65; *p* < 0.001) (Figure 9). Climate did not significantly affect the forage characteristics, grazing behaviors, livestock individual characteristics and the intake (*λ* = −0.04, 0.13 and 0.07, respectively; *p* > 0.05), but had a significantly negative effect on season (*λ* = −0.28; *p* < 0.05) (Figure 9). Forage characteristics had a significantly positive effect on grazing behavior (*λ* = 0.82; *p* < 0.001) and intake of yaks (*λ* = 0.50; *p* < 0.05), but did not significantly affect yak individual characteristics (*λ* = 0.06; *p* > 0.05) (Figure 9). Livestock individual characteristics significantly negatively affected grazing behaviors (*λ* = −0.19; *p* < 0.05) and the intake of yaks (*λ* = −0.31; *p* < 0.05) (Figure 9). We also found that compared with forage characteristics and grazing behaviors, only altitude had a strongly negative effect on yak intake (Figure 9).

## 4 Discussion

Grassland systems play pivotal roles in ecosystem services such as livestock products and health through livestock grazing (41–43). However, global grasslands are undergoing serious degradation due to climate change and land use (44–48). The alpine grasslands on the QTP have been continuously used as pasturelands for millennia by herders for grazing of livestock (e.g., yak and Tibetan sheep) (49). Numerous evidence has reported that the QTP’s grasslands have been overgrazed during past decades by rapidly increasing human and livestock populations (44). A direct consequence of grassland degradation is to lead to a decrease in pasture biomass and palatable grass species and consequently has a great impact on grazing behaviors of livestock (50, 51). In grassland ecosystems, plant communities often show patchy distribution patterns over space due to soil spatial heterogeneity and increasing disturbance of human and animals (50) or variations of an environmental gradient (e.g., altitude). Also, plant community composition and diversity can vary temporally (52), depending greatly on the differences in soil and climatic factors induced by season or short-term variability in precipitation and temperature (53–56). In this case, grazing behaviors of livestock will thus probably change at spatial–temporal scales with the shift in plant communities. We did find in the study that grazing behaviors of yak varied at both temporal and ranch scales relying on a visual observation in the fields, and importantly, intake of yaks was affected distinctly by multiple factors including climate, altitude, season, grazing behaviors, and forage and livestock individual characteristics based on a meta-analysis on the QTP. The findings verified our hypotheses about temporal and spatial variability in grazing behaviors of yaks and different factors that affecting yak intake at regional scales.

### 4.1 Temporal and spatial variations of grazing behavior

There were significant differences in grazing behaviors of yak including intake rates and walking speed within hours, among hours of each day and among days as well as across different observation sites. In this study, we only selected to carry out an observation for similar age of yaks, that is, the differences in individual characteristics such as the size and weight of yaks may be very small. Therefore, shifts in yak behaviors may result mainly from the differences in composition and biomass of plant communities and the nutrients of pasture in the local ranch or among ranches (44, 57). It is well known that plant communities are often found to have a mosaic distribution at different spatial scales (58). Even at a small local scale, the composition and biomass of palatable pasture may differ due to the effects of soil water content (54), grazing intensity (57, 59) and animal excrement (60). In fact, temporal variability in livestock behaviors has been reported in some previous studies. For example, several recent researches on the QTP have shown that grazing intensity may be the main factor affecting grazing behavior of yaks, and grazing behavior differed significantly between foraging time and distance traveled at light grazing (61, 62). Similarly, previous studies have reported that grazing season can affect heavily yak activities as well (63, 64). However, different from these reports, our findings provide evidence that even at a short temporal scale, such as within hours and among days, grazing behaviors of yaks also showed pronounced differences. In addition, we found that intake rate of yak was higher in the morning than in the afternoon. The finding might be related to the energy and metabolism of livestock. For instance, grazing yak generally grazing yaks generally need to acquire energy through daytime intake behavior after an overnight residence in the cattle sheds. The increased intake rate in the morning can be also explained by the fact that the ruminal pool is usually at its smallest in the morning due to the body’s natural process of expelling digesta (65, 66). The finding is consistent with prior reports in which showed that yak behaviors change with milking time and day/night cycles, which play major roles in the temporal distribution of intake rate (67, 68).

In contrast, we found a converse trend for walking speed in comparison to intake rate, that is, walking speed was relatively lower in the morning and higher in the afternoon. Such a discrepancy is reasonable because walking behavior generally increases energy consumption. Yaks need to save energy and allocate more time for foraging in the morning, but they commonly have higher rate of digestion through increasing walking and rumination in the afternoon. Actually, numerous studies have shown that forage acquisition is closely negatively correlated with walking speed in energy consumption of livestock (69–72). In addition to intake and walking behavior, rumination of yaks in the local ranch was mainly concentrated in the afternoon. The finding is in agreement with many previous studies showing that rumination activity in grazing yaks more frequently occurred in the afternoon (6, 33, 73, 74), probably because of the result of the high air temperature and solar radiation (7, 75).

### 4.2 The driving factors underlying the variations of yak intake based on meta-analysis

Despite observed temporal–spatial variability of grazing behaviors of yak, more focuses are needed to explore the consequence of varied grazing behaviors on foraging. For example, considering close associations between behaviors and feed intake of grazing livestock, we need clarify what factors affect livestock intake and which of them play direct or indirect roles in feed intake of livestock. As we can see from the Random Forest modeling base on a meta-analysis of the past 18-year dataset on the QTP, we indeed found that grazing behavior of yaks including resting, altitude, MAT, ASH, MAP, livestock age and season were the main predictors for yak intake in this area. The effects of these factors on yak intake have been reported in a recent study in which researchers found a close correlation between grazing behaviors of yaks and their intake (61). Some evidence has also shown that altitude would be an important predictor for grazing behaviors of yaks in summer if the pasture was utilized effectively (76). In addition, the influences of season and climate (e.g., MAP and MAT) on feed intake of yaks is likely to be related to the changes in plant communities induced by changed hydrothermal conditions over space and time. For example, yaks on the QTP are usually grazed relying on seasonal migrations between summer and winter pasture, with migrating to higher altitude pastures in summer and then moving back to lower altitude winter pastures in order to maintain the energy consumption (74, 77–79). This can be further supported by the results from structural equation model, showing that forage characteristics were affected by altitude and resulted in direct effects on grazing behaviors and intake of yaks. Individual characteristics of yaks were also found to directly affect their grazing behaviors and intake. This suggests that the differences in yak age, size and weight may lead to totally different amounts of grasses they eat.

### 4.3 Implications

Our findings confirm that grazing behaviors of yaks on the QTP can vary even at both small temporal scales and regional scales. Temporal and spatial variations of yak behaviors are strongly affected by the environments including climate, altitude, season and forage quality and biomass. This implies that multiple factors can be responsible for the variations in livestock behaviors and shifts in behavioral patterns may consequently lead to positive or negative feedback to QTP’s grassland ecosystems through plant–animal interactions. Future work could focus on sustainable grassland management via modulating behaviors of grazing livestock on the QTP.

## 5 Conclusion

Altogether, we provide evidence that grazing behaviors of yaks on the QTP varied at small temporal and regional scales, and multiple factors involved in climate, altitude, season, forage and individual characteristics can have an effect on yak intake. Admittedly, some research limitations might exist in this study as only a visual observation was conducted for monitoring grazing behaviors of yaks, although a direct observation is generally reckoned as one of an effective way to investigate animal behaviors. It is necessary that more technologies such as developed wearable wireless sensors for continuously monitoring eating, rumination, laying and body temperature etc. in combination to visual observation should be widely utilized in the future studies, to provide more accurate parameters for modeling the relationships between grazing livestock and forages in rangeland ecosystems.

## Data availability statement

The original contributions presented in the study are included in the article/Supplementary material, further inquiries can be directed to the corresponding author.

## Author contributions

XY: Data curation, Formal analysis, Funding acquisition, Investigation, Methodology, Project administration, Resources, Software, Writing – original draft. UD: Funding acquisition, Investigation, Methodology, Project administration, Resources, Supervision, Writing-original draft, Writing – review & editing. JM: Data curation, Investigation, Writing – review & editing. XL: Data curation, Investigation, Writing – review & editing. QF: Data curation, Investigation, Writing – review & editing. HZ: Data curation, Investigation, Writing – review & editing. X-BW: Conceptualization, Formal analysis, Funding acquisition, Investigation, Project administration, Resources, Supervision, Software, Writing – review & editing.

## Footnotes

^1^http://gzhatu.com/du2dfm.html

^2^https://apps.automeris.io/wpd/index.zh_cn.html
