# Supplementary material for: Temporal–spatial variability of grazing behaviors of yaks and the drivers of their intake on the eastern Qinghai-Tibetan Plateau
Source: Front Vet Sci. 2024 Jun 11;11:1393136. doi: 10.3389/fvets.2024.1393136 (PMC11197466; doi:10.3389/fvets.2024.1393136)
Supplement: Supplementary file 2 [file Image_1.pdf]

Figure S1. Variations in intake rate (bites/min) of yaks across observation days. Red and green bars with standard errors indicate the intake rate (bites/min) of yaks observed in the morning (AM) and in the afternoon (PM), respectively. The statistically significance was tested by ANOVA at $P < 0.05$.

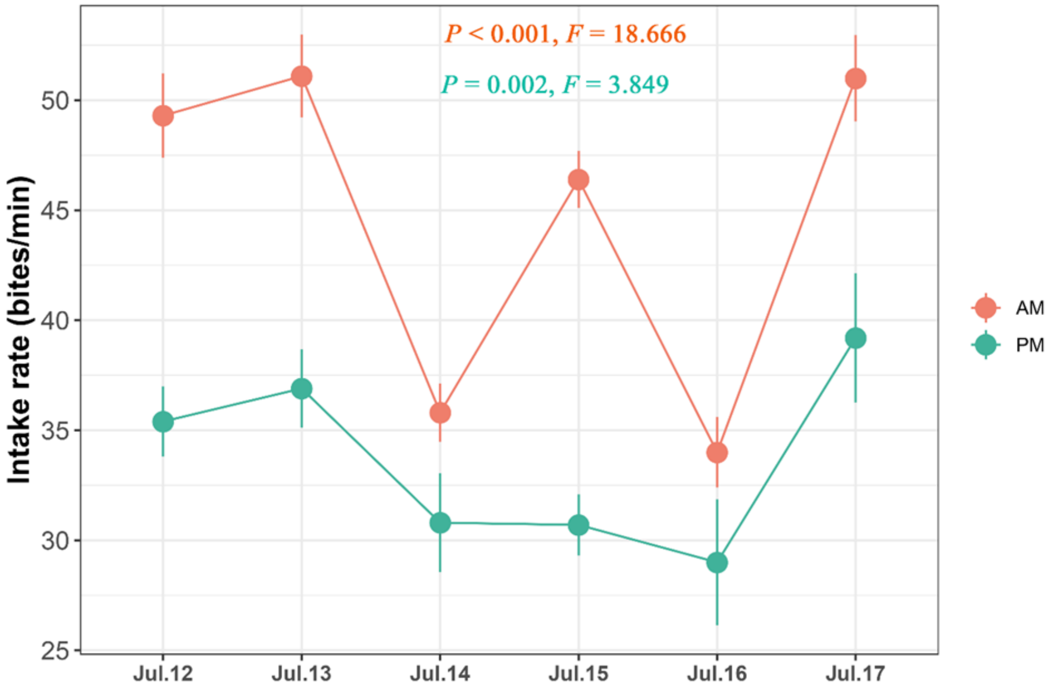

Figure S2. Variations in walking speed (steps/min) of yaks across observation days. Red and green bars with standard errors indicate the walking speed (steps/min) of yaks observed in the morning (AM) and in the afternoon (PM), respectively. The statistically significance was tested by ANOVA at $P < 0.05$.

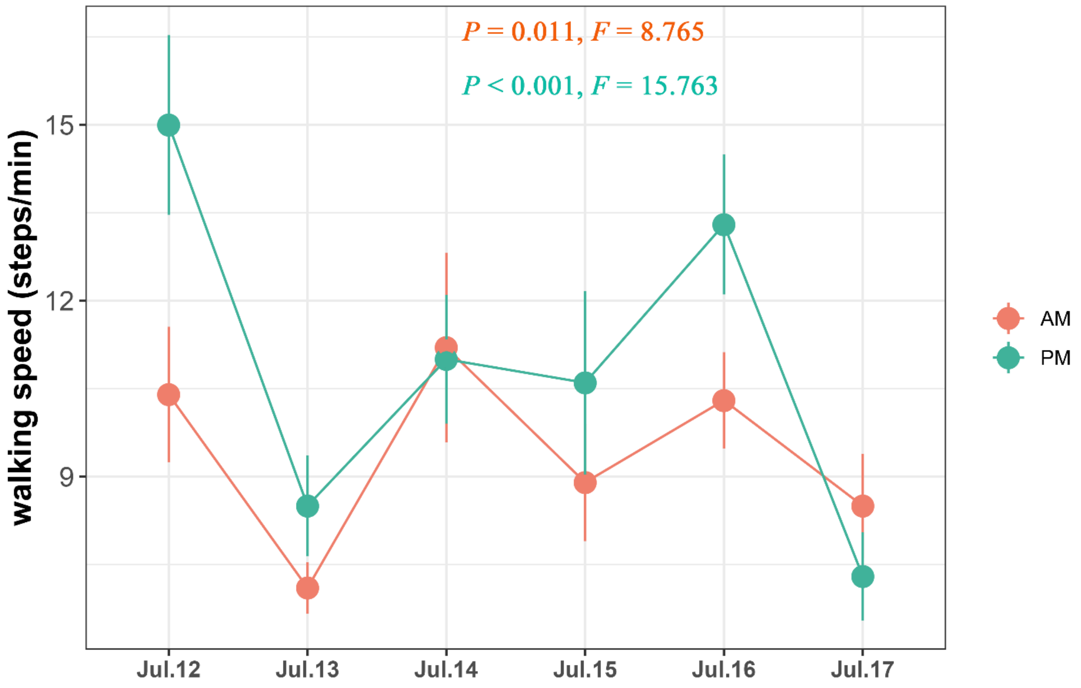

Figure S3. Flow Diagram reporting of literature search and selection process.

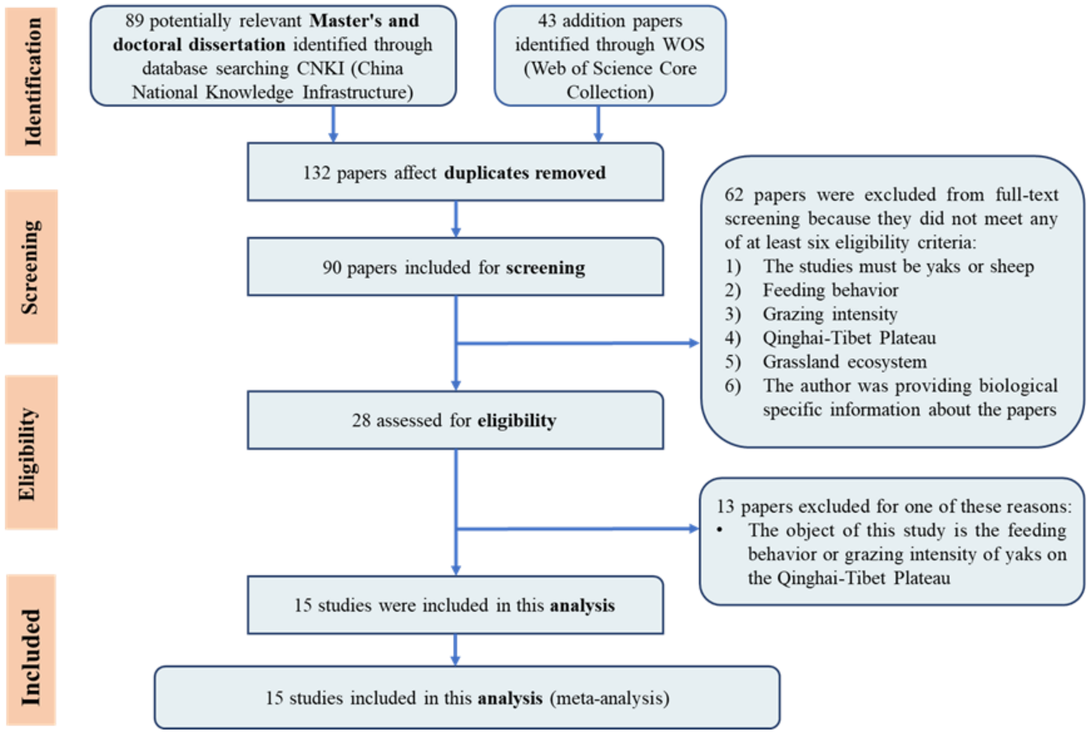

Figure S4. Violin diagram of the differences in Walking speed (/min) and Rumination (/min) of yaks across different observation sites of Qinghai-Tibetan Plateau.

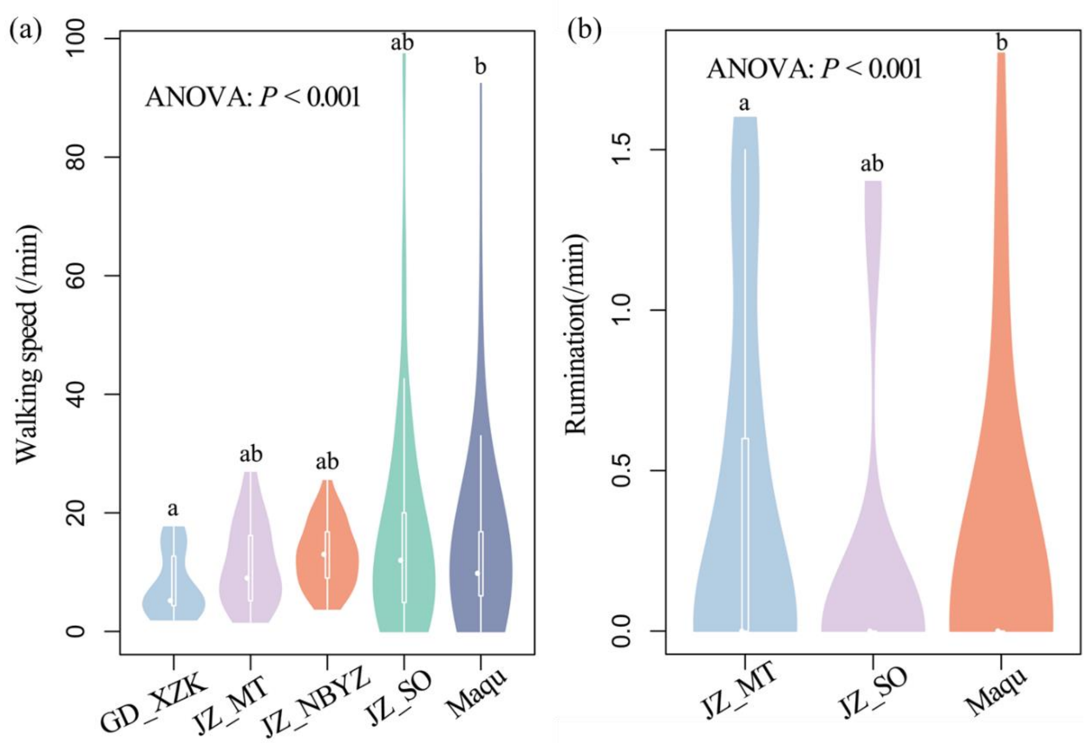

Figure S5. The normalization distribution test for all the data we used for ANOVA.

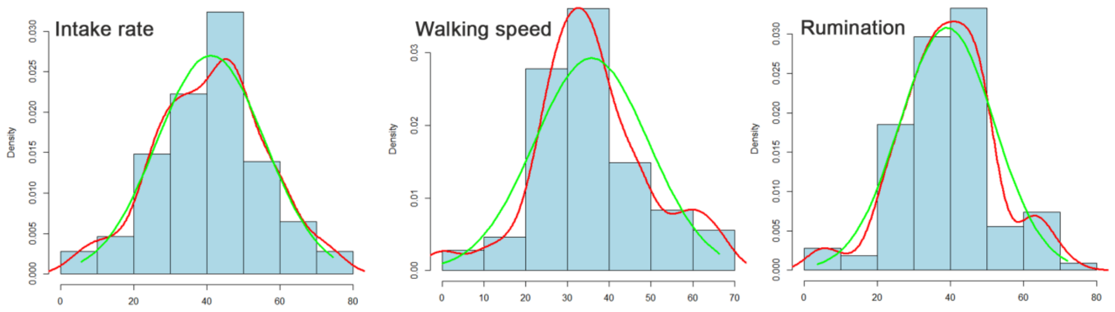

Table S1. The reference information we selected for meta-analysis.

Number	References	First author	Publication date	Website link
1	Effects of Seasonal Grazing on The Relationship Between Alpine Meadow and yak	Youshun Jin	2022	https://www.cnki.net
2	Seasonal Changes in Grazing Behavior and Rumen Microbiota of Yak and Cattle	Chen Li	2020	https://www.cnki.net
3	Effects of Shrub Encroachment on Grazing Behavior, Rumen Fermentation and Serum Parameters of Yaks in Alpine Meadows	Chuntao Yang	2021	https://www.cnki.net
4	Effects of Cold and Warm Season on Grazing Behavior and Rumen Microbial Diversity of Free-rang yak	Chen Li	2020	https://www.cnki.net
5	Study on yak's grazing behavior in summer and winter pasture using GPS and GIS technology	Yanfu Bai	2017	https://www.cnki.net
6	Study on grazing behavior of yak and cattle-yak in autumn pasture of Qilian mountain by GPS tracking and positioning system	Peipei Liu	2015	https://www.cnki.net
7	Effects of different grazing intensities on grazing behavior of yak in summer	Fayang Liu	2009	https://www.cnki.net
8	Study on Grazing Behaviour of Yaks in Summer, Autumn and Winter	Luming Ding	2007	https://www.cnki.net
9	Effects of Grazing Regime on Feed Intake and Apparent Digestibility of Yak and Tibetan Sheep	Yanfen Zhang	2020	https://www.cnki.net

Number	References	First author	Publication date	Website link
10	Botanical composition and grazing behavior of Qinghai yaks of Plateau type in the natural rangeland	Rende Song	2008	https://www.cnki.net
11	Shrub cover impacts on yak growth performance and herbaceous forage quality on the Qinghai-Tibet Plateau, China	Chuntao Yang	2021	https://www.webofscience.com
12	Behavioral patterns of yaks (Bos grunniens) grazing on alpine shrub meadows of the Qinghai-Tibetan Plateau	Chuntao Yang	2021	https://www.webofscience.com
13	Behavioral characteristics of yaks grazing summer and winter pastures on the Qinghai-Tibetan Plateau	Peipei Liu	2021	https://www.webofscience.com
14	Seasonal heat production and energy balance of grazing yaks on the Qinghai-Tibetan plateau	Luming Ding	2014	https://www.webofscience.com
15	Grazing Behavior of Lactating and Non-Lactating Yaks in the Summer Season of the Qinghai-Tibetan Plateau	Luming Ding	2006	https://www.webofscience.com
